# EXAMINING EXCLUSION FOR GUIDANCE TO INCLUSION: A GOOD IDEA?

**DOI:** 10.1093/geroni/igac059.1516

**Published:** 2022-12-20

**Authors:** Lyn Holley

**Affiliations:** University of Nebraska at Omaha, Omaha, Nebraska, United States

## Abstract

This paper is based on a literature review that informed writing of a chapter for a new edited book, Moving beyond the rhetoric - research with older service users – why ethics and integrity matter. (Emerald, in review). This literature review was undertaken to ground guidance for the chapter in the book that addresses ethics in research with American Indians of all tribes on lands currently part of the USA. A theme that emerged uninvited was the many ways in which inquiry that was unintentionally or deliberately unethical also operated to reduce inclusion of American Indians in research – often the very research needed to discern or demonstrate value of various clinical, social, or other types of interventions needed to improve policies or practices. This paper explores that theme and its implications for research with American Indians.

